# Muscarinic acetylcholine receptors M_2_ are upregulated in the atrioventricular nodal tract in horses with a high burden of second-degree atrioventricular block

**DOI:** 10.3389/fcvm.2023.1102164

**Published:** 2023-11-16

**Authors:** Sarah Dalgas Nissen, Arnela Saljic, Helena Carstensen, Thomas Hartig Braunstein, Eva Melis Hesselkilde, Sofie Troest Kjeldsen, Charlotte Hopster-Iversen, Alicia D’Souza, Thomas Jespersen, Rikke Buhl

**Affiliations:** ^1^ Laboratory of Cardiac Physiology, Department of Biomedical Sciences, Faculty of Health and Medical Sciences, University of Copenhagen, Copenhagen, Denmark; ^2^ Institute of Pharmacology, West German Heart and Vascular Centre, University Duisburg-Essen, Essen, Germany; ^3^ Department of Veterinary Clinical Sciences, Faculty of Health and Medical Sciences, University of Copenhagen, Taastrup, Denmark; ^4^ Core Facility for Integrated Microscopy, Department of Biomedical Sciences, Faculty of Health and Medical Sciences, University of Copenhagen, Copenhagen, Denmark; ^5^ Division of Cardiovascular Sciences, University of Manchester, 3.30 Core Technology, Manchester, United Kingdom

**Keywords:** second-degree AV block, cardiac conduction, parasympathetic activity, vagus, acetylcholine-dependent inwardly rectifying potassium channel, muscarinic receptor, equine, atrioventricular node

## Abstract

**Background:**

Second-degree atrioventricular (AV) block at rest is very common in horses. The underlying molecular mechanisms are unexplored, but commonly attributed to high vagal tone.

**Aim:**

To assess whether AV block in horses is due to altered expression of the effectors of vagal signalling in the AV node, with specific emphasis on the muscarinic acetylcholine receptor (M_2_) and the G protein-gated inwardly rectifying K^+^ (GIRK4) channel that mediates the cardiac *I*_K,ACh_ current.

**Method:**

Eighteen horses with a low burden of second-degree AV block (median 8 block per 20 h, IQR: 32 per 20 h) were assigned to the **control group**, while 17 horses with a high burden of second-degree AV block (median: 408 block per 20 h, IQR: 1,436 per 20 h) were assigned to the **AV block group**. Radiotelemetry ECG recordings were performed to assess PR interval and incidence of second-degree AV block episodes at baseline and on pharmacological blockade of the autonomic nervous system (ANS). Wenckebach cycle length was measured by intracardiac pacing (*n* = 16). Furthermore, the expression levels of the M_2_ receptor and the GIRK4 subunit of the I_KACh_ channel were quantified in biopsies from the right atrium, the AV node and right ventricle using immunohistochemistry and machine learning-based automated segmentation analysis (*n* = 9 + 9).

**Results:**

The AV block group had a significantly longer PR interval (mean ± SD, 0.40 ± 0.05 s; *p* < 0.001) and a longer Wenckebach cycle length (mean ± SD, 995 ± 86 ms; *p* = 0.007) at baseline. After blocking the ANS, all second-degree AV block episodes were abolished, and the difference in PR interval disappered (*p* = 0.80). The AV block group had significantly higher expression of the M_2_ receptor (*p* = 0.02), but not the GIRK4 (*p* = 0.25) in the AV node compared to the control group. Both M_2_ and GIRK4 were highly expressed in the AV node and less expressed in the atria and the ventricles.

**Conclusion:**

Here, we demonstrate the involvement of the m_2_R-*I*_K,ACh_ pathway in underlying second-degree AV block in horses. The high expression level of the M_2_ receptor may be responsible for the high burden of second-degree AV blocks seen in some horses.

## Introduction

Second-degree atrioventricular (AV) block is frequently recognized in horses and is reported to be the most common cardiac arrhythmia occurring in 13%–40% of horses across breeds (1–4). Recently, however, we showed that almost 90% of the Standardbred racehorses display second-degree AV block when studying 24-hour Holter ECG recordings (5). This could imply that second-degree AV blocks in horses are much more common than previously reported, or that the phenomenon varies between equine breeds. Nevertheless, despite being such a frequent arrhythmia among these animals, little is known about the underlying mechanism.

Second-degree AV blocks are defined as the loss of 1:1 conduction of atrial impulses to the ventricles, which in the ECG, is represented by individual P-waves not followed by a QRS complex (6). Second-degree AV block has been associated with decreased performance, while myocarditis along with varying degrees of degenerative changes and inflammation in the AV node have been suggested to induce advanced second-degree AV block in horses (7, 8). However, most commonly, the second-degree AV blocks found in horses are considered a normal physiological trait, strongly influenced by vagal activity (2, 9). By definition, second-degree AV blocks are supposed to be harmless if they disappear when parasympathetic activity is withdrawn, e.g., during exercise (10, 11). However, as an enormous variation in the number of second-degree AV block episodes exists among horses, the assessment of the clinical significance of the pronounced presence of second-degree AV block is rather challenging and accurate exclusion of dysfunction of the AV node is difficult to determine. Furthermore, as horses develop exercise-induced cardiac remodeling and as a species suffer from atrial fibrillation like humans, these animals have gained interest as large animal research models for cardiac arrhythmias (5, 12–16). Therefore, more knowledge on the cardiac conduction in horses and the underlying mechanism of second-degree AV block in otherwise healthy horses is warranted.

Second-degree AV blocks are reported to occur mainly when horses are at rest and are often accompanied by sinus arrhythmia and fluctuations in the duration of PR interval (2, 17). In most cases, the AV blocks disappear once the horses exercise, emphasizing that parasympathetic activity may be a prerequisite in the genesis of second-degree AV block. This has previously been supported by studies where both interruption of the left vagal nerve and pharmacological blocking of the parasympathetic activity lowered or completely abolished the presence of second-degree AV block (15, 17). It has been suggested that the second-degree AV blocks were related to fluctuations in blood pressure, creating a feedback mechanism via the aortic arch and the carotid sinus activating parasympathetic activity (7). However, this association is highly inconsistent with the results (17), and does not explain why some horses exhibit numerous of second-degree AV block episodes, while others do not exhibit even a singular block. Yamaya and colleagues, who investigated the AV refractoriness before and after pharmacological blocking of the autonomic nervous system (ANS), suggested that an intrinsic, but yet unexplored, component must exist in horses with a high burden of second-degree AV blocks, and argued that this could be the explanation for the large differences in phenotypes between horses (18).

**Figure 1:**
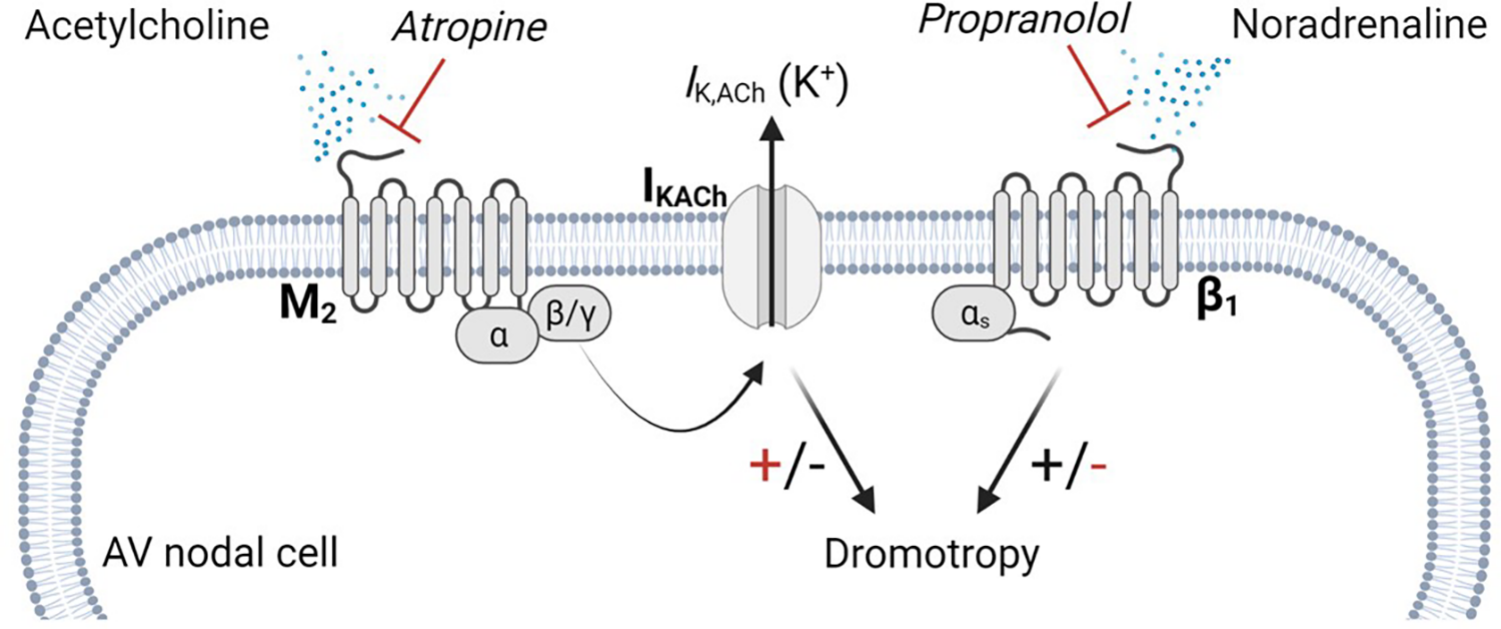
m_2_R-*I*_K,ACh_ pathway. Parasympathetic modulation on cardiac myocytes are primarily carried by the m_2_R-*I*_K,ACh_ pathway. Acetylcholine released from the vagal nerve activates the muscarinic M_2_ receptor, which is a G-protein coupled receptor. Upon activation, the G-protein dissembles and the β/γ subunit activates the I_KACh_ channel, facilitating an efflux of potassium. Atropine antagonizes the binding of acetylcholine on the M_2_ receptor, which lowers I_KACh_ channel open probability, resulting in a positive dromotropic effect, whereas propranolol inhibits the positive dromotropic effect of norepinephrine on AV nodal cells. AV, Atrioventricular.

A key pathway that is potentially involved in the genesis of second-degree AV block, is the m_2_R-*I*_K,ACh_ pathway, which consists of the muscarinic acetylcholine receptor (M_2_) and the acetylcholine-dependent inwardly rectifying potassium channel (I_KACh_) (19). This pathway is relevant, as parasympathetic modulation on the heart, is primarily mediated by acetylcholine (ACh) release from the vagal nerve. Acetylcholine binds to the M_2_ receptor, which initiates several intracellular cascades, including the disassembly of the G protein, whereby the βγ subunit binds to the I_KACh_ channel, facilitating the open probability and potassium efflux (20) (Figure 1). Furthermore, this pathway has been shown to be involved in the genesis of AV block in mammals (21). The potassium efflux results in hyperpolarisation of the membrane potential breaking the phase 0 and phase 4 depolarisation of the automated cells to a degree where the conduction of the electrical signal is delayed or even lost (22). Isolated Guinea pig hearts stimulated with ACh displayed complete AV block, while tertiapin, a selective I_KACh_ blocker, dose-dependently reversed the ACh-induced AV block (21). We previously showed that by blocking the I_KACh_ channel in horses, the PR interval shortens and atrial to ventricular conduction increases, suggesting a central role of this channel in second-degree AV block origin in horses (14). However, the mechanism of second-degree AV block in horses remains unclear. Here, we investigated the role of parasympathetic activity on the genesis of second-degree AV block in horses, focusing on the local expression of the M_2_ receptors and the I_KACh_ channels.

## Material and methods

### Animal experiments

The study was performed in accordance with the European Commission Directive 86/609/EEC after obtaining approval from the local ethical committee at the Department of Veterinary Clinical Sciences, University of Copenhagen and the Danish Animal Experiments Inspectorate under the license number 2016-15-0201-01128.

Thirty-five Standardbred racehorses (16 geldings and 19 mares, mean age 7.1 years (range 4–13 years), mean body weight (BW) 489 kg (range 404–609 kg)) were included in the study. The horses were divided into two groups according to the presence of second-degree AV block episodes during their 24-hour Holter ECG recording (KRUTECH Televet, Kruuse A/S, Maarslev, Denmark). Horses with very few or no AV block episodes were assigned to the **control group**, whereas horses presenting with an intermediate to high burden of AV block (>100 AV block episodes) were assigned to the **AV block group**. Throughout all the subsequent data analysis, the authors were kept blinded to the AV block status of the horses. Next, the PR interval and number of second-degree AV block episodes were assessed in the horses before and after blocking their autonomic nervous system (ANS). Twenty-two of the 35 horses were included in an electrophysiological (EP) study determining their Wenckebach cycle length, a measure of AV nodal refractoriness. Finally, the horses were euthanized and biopsies encompassing the AV node were collected for the assessment of the expression of the M_2_ receptor and the I_KACh_ channel. Please, refer to the Supplementary Material for further information on the inclusion process and existing use of data from the horses.

### ECG analysis and block of the autonomic nervous system

The number of second-degree AV block episodes was counted from the Holter ECG recording (20 h) using Televet100 software version 6.0.0. For baseline resting ECG analysis, twenty consecutive beats, without arrhythmias including second-degree AV blocks, from the 24-hour ECG recordings were chosen and imported to LabChart 8 (v8.1.16, ADInstruments) where the PR interval was measured manually. Within a week from the baseline recordings, the measurements were repeated five minutes after the IV injection of atropine (Atropine sulphate 10 mg/ml; 0.04 mg/kg) and propranolol (Propranolol hydrochloride 10 mg/ml; 0.2 mg/kg), which have been shown to induce complete blocking of the ANS (15, 23). Atropine blocks the M_2_ receptor and thus constitutes a downstream decrease in the open probability of the I_KACh_, thereby blocking the effect of the parasympathetic limb of the ANS (Figure 1). To avoid unknown effects of sympathetic activity on AV nodal conduction, in the absence of parasympathetic activity, propranolol which blocks β-receptors was also administered (Figure 1). Before injection of atropine and propranolol, a 12G catheter [IntraflonTM 2 (PTFE). 12G. Ecouen, France] was placed in the jugular vein and the horse equipped with a base-apex 3-lead ECG. The horses were placed in their stall where hay and feed had been removed, but water intake was allowed. The lights were turned off and the surroundings kept quite during the procedure, which lasted ∼5–10 min, followed by two hours of rest until the effect of atropine ceased. First, propranolol was infused as a bolus, which was followed by flushing with saline and the immediate infusion of atropine, followed by a subsequent saline flushing. The horses were observed during the entire procedure and during the two hours of rest that followed. One hour after ANS blocking, the horses received an IV dose of flunixin-meglumin (finadyne®vet MSD, Segré, France, 1.1 mg/kg) for the relief of any discomfort.

### Wenckebach cycle length

Wenckebach cycle length was assessed in 22 of the 35 horses during general anesthesia, who had been included in a former study exploring training-induced atrial remodeling (24). During open-chest surgery a high-density epicardial multi-electrode device was placed on the right atrium (RA) and used to generate extra stimulus protocols to obtain functional data as previously described (25). Briefly, Wenckebach cycle length was measured by atrial pacing at 1,200 ms and sequentially shortening the pacing cycle length (PCL) with 20 ms at every 20th beat until Wenckebach point was detected (1:1 atrial to ventricular conduction lost). The measurement was repeated three times and was successful in 16 horses. Due to time restrictions during the procedure Wenckebach cycle length was not measured in the remaining six horses.

### Tissue analysis

**Figure 2:**
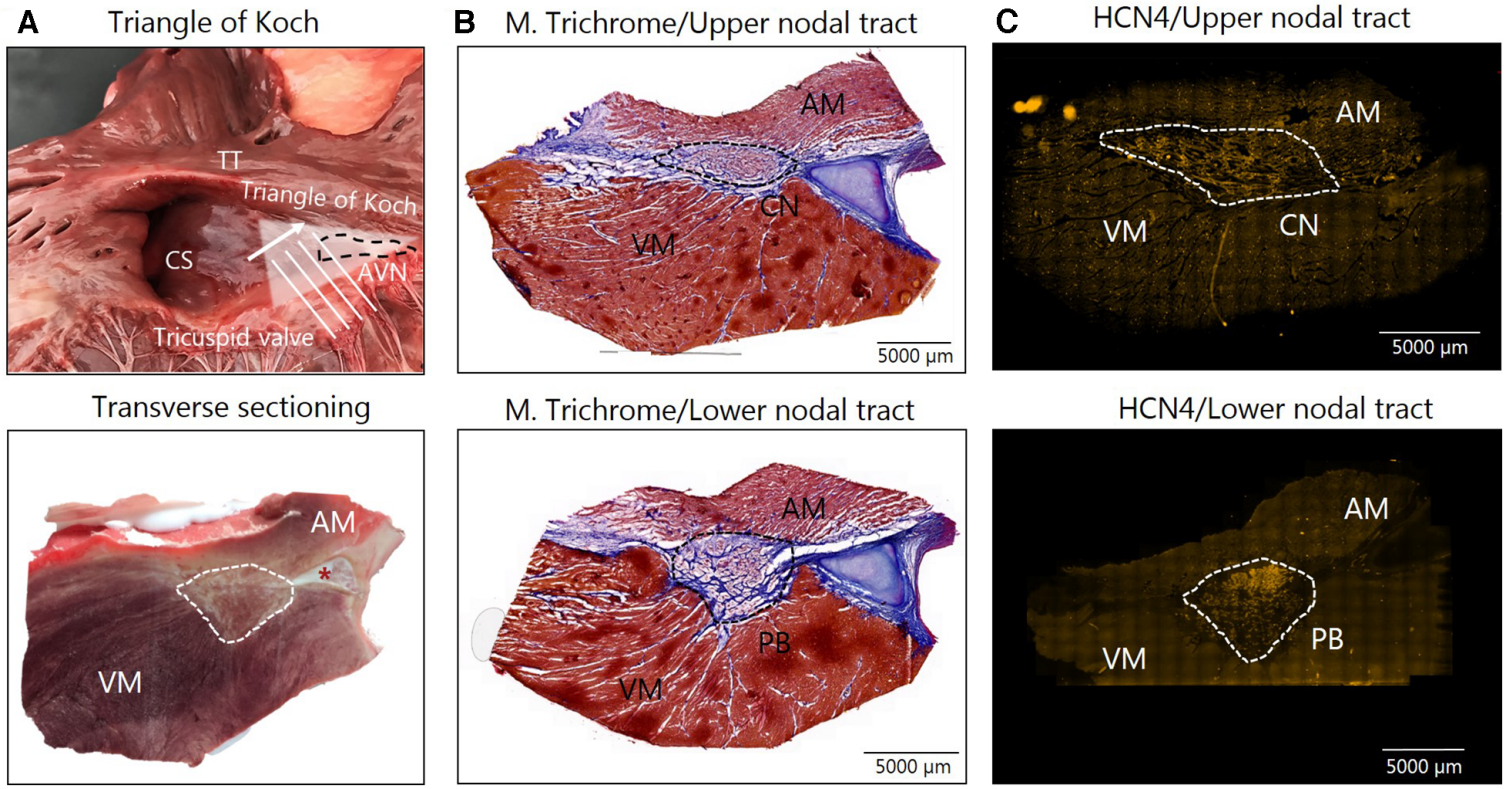
Av nodal sampling. (**A**) Tissue samples form the right atria at the tip of the triangle of Koch (top) were snap frozen and transverse cryosectioning of the AV node in the direction of the white arrow was performed, which is illustrated in the bottom image. (**B**) Masson's Trichome staining of samples from the CN (top) and PB (bottom) was followed by immunohistochemical labeling of HCN4 for the identification of the AV node (**C**) In the Masson's Trichome images, myocytes are stained red/bordeaux and fibrosis blue. An asterisk marks the aortic valve base of the non-coronary leaflet, which is cartilaginous in some horses. AM, atrial myocardium; AVN, Atrioventricular node; CN, Compact node; CS, Coronary sinus; HCN4, hyperpolarized cyclic nucleotide–gated channel 4; M. Trichome, Masson's Trichome; PB, Penetrating bundle; VM, Ventricular myocardium.

Nine horses in each group were sacrificed, the hearts removed and biopsies encompassing the AV node were harvested within 30 min. The *triangle of Koch* in the RA was identified, and the compact node and the penetrating bundle were excised as described previously (15) (Figure 2A). The tissue samples were immediately frozen in isopentane mixed with dry ice. Samples were stored in a freezer at −80°C until further processing. Tissue blocks were cryosectioned perpendicular to the AV node (Figure 2A). Sections, 30 *µ*m thick, were cut and collected onto glass slides (Superfrost plus, VWR International ltd). Histological staining with Masson's Trichome staining and immunolabeling of the hyperpolarization activated cyclic nucleotide–gated channel 4 (HCN4) specific for nodal tissues was performed to identify and confirm correct sampling of the AV node as previously described (15). Once the correct location of the AV conduction axis was confirmed via HCN4 positive staining, a sample from the upper nodal tract corresponding to the compact node (CN), consisting of loosely packed HCN4 positive cells on the atrial side of the central fibrous body was chosen for analysis. Furthermore, samples from the lower nodal tract, corresponding to the penetrating bundle (PB), consisting of HCN4 positive cells completely encapsulated in the central fibrous body were also chosen for analysis (Figures 2B,C). The samples were prepared for the analysis of expression of M_2_ receptor and for GIRK4, one of the two subunits of the I_KACh_ channel. Both subunits GIRK1 and GIRK4 are believed to be central in forming the I_KACh_ in the heart (26, 27). Targeting GIRK4 was chosen, as the commercial availability of specific antibodies of GIRK1 was limited. For M_2_ receptor immunolabeling, samples were fixed in 10% neutralized formalin for 30 min, followed by three times 10 min washes in phosphate-buffered saline (PBS). Sections were permeabilized with 0.1% Triton X-100 (Sigma-Aldrich, Cat. No. X-100) for 30 min, again followed by three washes in PBS. Blocking was performed for one hour in 1% Bovine Serum Albumin (BSA) in PBS at room temperature. Incubation with primary antibodies (rabbit Anti-CHRM2 Antibody, #AMR-002, 1:100, Alomone Labs) diluted in 1% BSA was carried out at 4°C over night. For GIRK4 immunolabeling, tissue was fixed in pre-cooled acetone (−20°C) for 10 min followed by 30 min blocking and permeabilization in 0.2% Triton X-100 along with fish skin gelatine in PBS. The samples were then incubated in primary antibodies [rabbit anti-KCNJ5 (K_ir3.4_)/GIRK4, APC-027, 1:100, Alomone Labs] diluted in the same buffer overnight at 4°C. For both M_2_ and GIRK4 secondary antibodies (Alexa Fluor®568-conjugated goat anti-rabbit IgG (1:500, Life Technologies) in 1% BSA was applied for 90 min at room temperature. After a final three washes in PBS, sections were mounted in VECTASHIELD antifade mounting media (Novus, Cat. No. H-1000) for M_2_ receptor, and in Prolong Gold Diamond (Life Technologies) for GIRK4. One slide without primary antibody was processed as a negative control for both. Images were acquired using a Zeiss AxioScanner Z1, a fluorescence microscope combined with Axiocam MRm at 20x magnification using the appropriate filters. Microscopic settings were kept identical for all samples. The images were imported into ZEN 3.5 Blue edition (Carl Zeiss AG) and segmented using the extension software ZEN Intellesis Trainable Segmentation (Carl Zeiss AG, 2017). The machine learning pixel classifier (33 features) helped to segment the images into three distinctly defined classes: “cardiac myocytes”, “collagen” and “background”. During the training of the machine-learning algorithm, the operator ensured that the images selected for training represented the biggest variety within the dataset, to make sure the machine-learning algorithm would be a valid generalized algorithm capable of handling all images in the dataset. The training of a machine-learning algorithm is done iteratively and with the trainer capable of judging the different classes to be able to correct the algorithm through the process. The end goal is to get the algorithm to perform as good as any well-trained human operator, while being more persistent and uniform in its performance. After analysis of the segmented areas, the expression of M_2_ receptor and GIRK4 within the AV node, the atrium and the ventricle could be quantified. From this, the mean intensity of M_2_ and GIRK4 expression and the total area of collagen was quantified. A similar separate model was created for each of the proteins of interest. We based the analysis on an average expression throughout the entire AV nodal axis. Furthermore, the expression level from the upper nodal tract, the CN, and the lower nodal tract, the PB, was assessed, only including samples where the CN and PB could be clearly distinguished from one another (Figures 2B,C). However, nine of the horses had been included in a previous study (15), and only samples from either the CN or the PB were left for analysis. For the M_2_ receptor analysis, only samples from eight horses in each group were left for staining and analysis. Besides analysing the expression in the AV node, a region in both the RA and in the ventricular septum were also analyzed. As the expression of GIRK4 turned out to be unexpectedly high in the ventricular tissue, a ventricular region remote to the AV node (more than 0.5 cm distally) was also analyzed.

### Statistics

Statistical analysis was performed using GraphPad Prism 8 software (GrapPad Software, San Diego, CA). Normality was assessed using the Shapiro-Wilk test. All data are presented as mean and standard deviation (SD). The statistical methods used, were unpaired *t*-tests and Mann-Whitney *U*-test for ECG parameters. For M_2_ and GIRK4 protein expression in the AV node a *t*-test was used, while an ordinary one-way ANOVA was used to explore the expression level between the atrium, AV node and ventricle. Lastly, a paired *t*-test was used for the comparison of GIRK4 expression in the two ventricular regions. A two-sided *p* value ≤_ _0.05 was considered statistically significant. Linear regression was performed for identification of any correlation between the number of second-degree AV block and the expression of M_2_ and GIRK4 in the AV node.

## Results

### Study groups

The control group consisted of 18 horses (9 geldings and 9 mares) mean age: 6.7 ± 1.8 years, mean BW: 497 ± 39 kg. The AV block group consisted of 17 horses (6 geldings and 11 mares) mean age: 7.4 ± 2.4 years, mean BW: 480 ± 58 kg.

**Figure 3:**
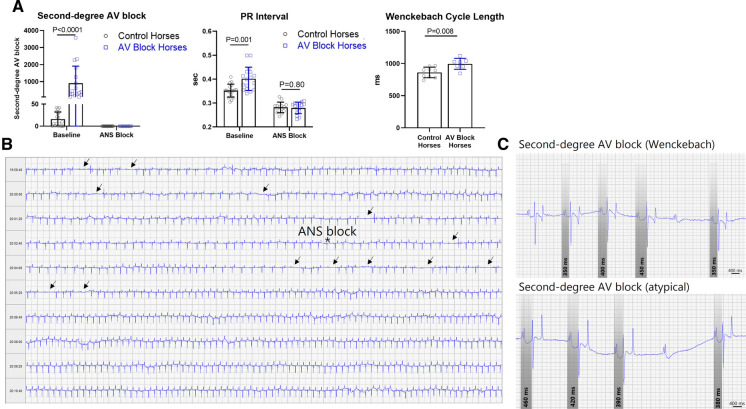
Second-degree AV block and PR interval. (**A**) The group consisting of the AV block horses had significantly more second-degree AV block, longer PR interval and Wenckebach cycle length compared to the control group. Pharmacological block of the ANS abolished second-degree AV block in all horses and diminished the difference in PR interval. The data is presented as mean ± SD. (**B**) Representative of an ECG recording of a horse exhibiting massive numbers of second-degree AV block (arrows) prior to blockade of the ANS marked by an asterisk. (**C**) Examples of the classical Wenckebach type second-degree AV block with progressive prolongation of the PR interval prior to the blocked beat and bottom an example of atypical varying PR interval pattern prior to the blocked beat, also very common in horses. ANS, Autonomic nervous system; AV, Atrioventricular.

No difference in age (*p* = 0.30) and BW (*p* = 0.31) was found between groups. The control group presented with median second-degree AV block episodes: 8/20 h (IQR: 32/20 h) whereas the AV block horses presented with median second-degree AV block episodes: 408/20 h (IQR: 1,436/20 h) being significantly higher (*p* < 0.0001) (Figure 3A).

### AV nodal conduction

As expected, the PR interval was significantly longer in the AV block group compared to the control group (*p* = 0.001), as was the Wenckebach cycle length (*p* = 0.008). Blocking of the ANS abolished second-degree AV blocks and diminished the difference in PR interval between the two groups (Figures 3A,B). Representative examples of a Wenckebach second-degree AV block along with an atypical PR interval pattern prior to AV block are displayed in Figure 3C. All horses with second-degree AV block presented with mixed PR interval patterns before AV block. Among these cases, progressive prolongation of the PR interval prior to the blocked beat was the dominating circumstance, but not the exclusive condition. This type of second-degree AV block is recognized as the Wenckebach type in human cardiology.

### Expression of the M_2_ receptor and GIRK4

**Figure 4:**
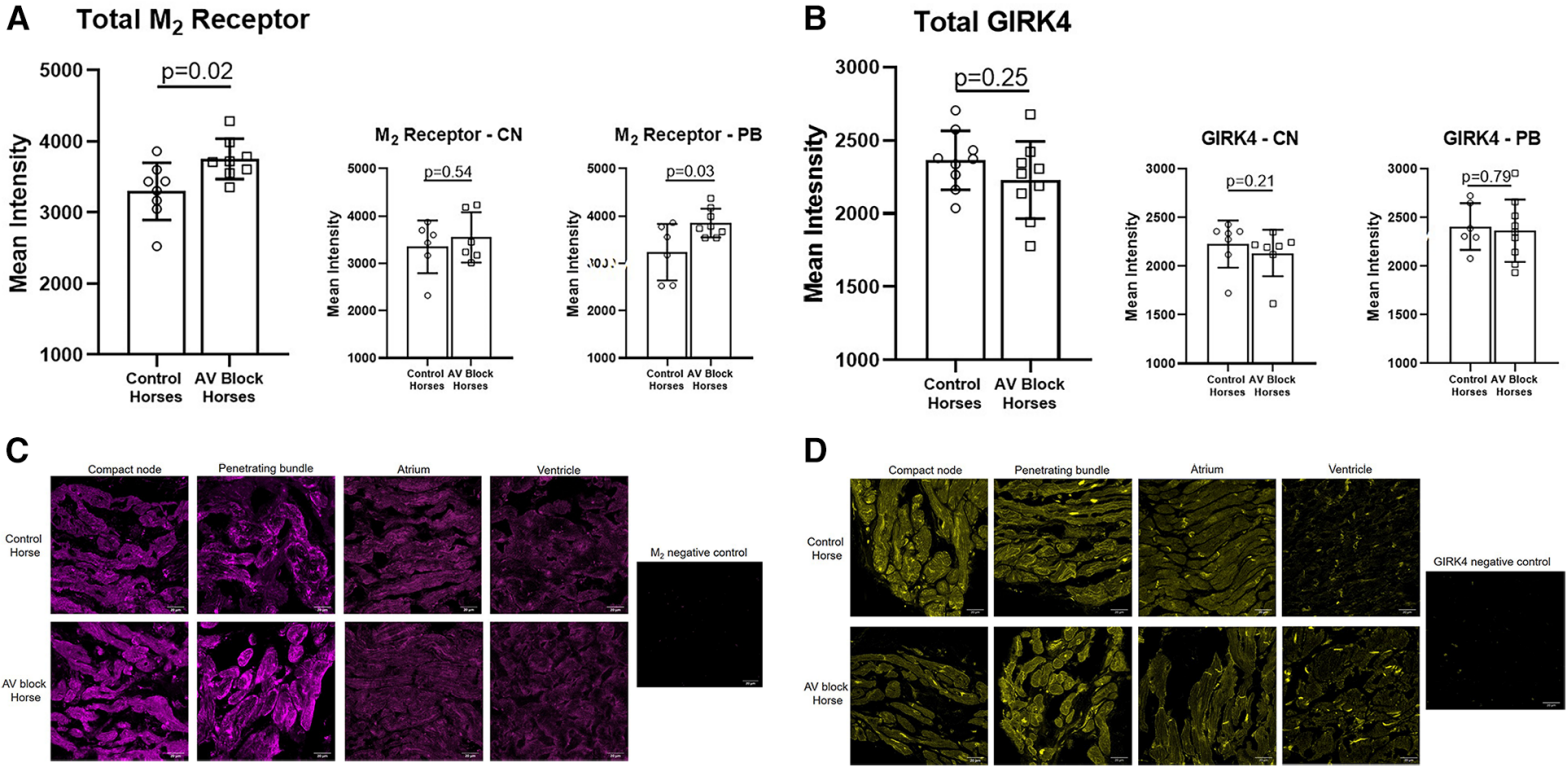
Av nodal expression of the M_2_ receptor and the GIRK4 channel. (**A**) A significantly higher expression of the M_2_ receptor was found in the PB (*n* = 6 + 8) and in total AV nodal (*n* = 8 + 8) expression in the AV block horses. (**B**) No difference in the expression of GIRK4 was found between groups (*n* = 9 + 9). Representative examples of immunohistochemical staining of the M_2_ receptor (**C**) and GIRK4 (**D**) in the CN, PB, atrium and ventricle along with a negative control for both. Note the difference in ion channel localization for especially GIRK4, which in the atrium and ventricle is highly expressed in the intercalated discs. Intercalated discs are underdeveloped in the AV nodal cells (28), wich explains why a more diffuse expression is present. The images were acquired on a confocal microscope (LSM700, Zeiss, magnification: 40x). AV, Atrioventricular; CN, Compact node; PB, Penetrating bundle.

**Figure 5:**
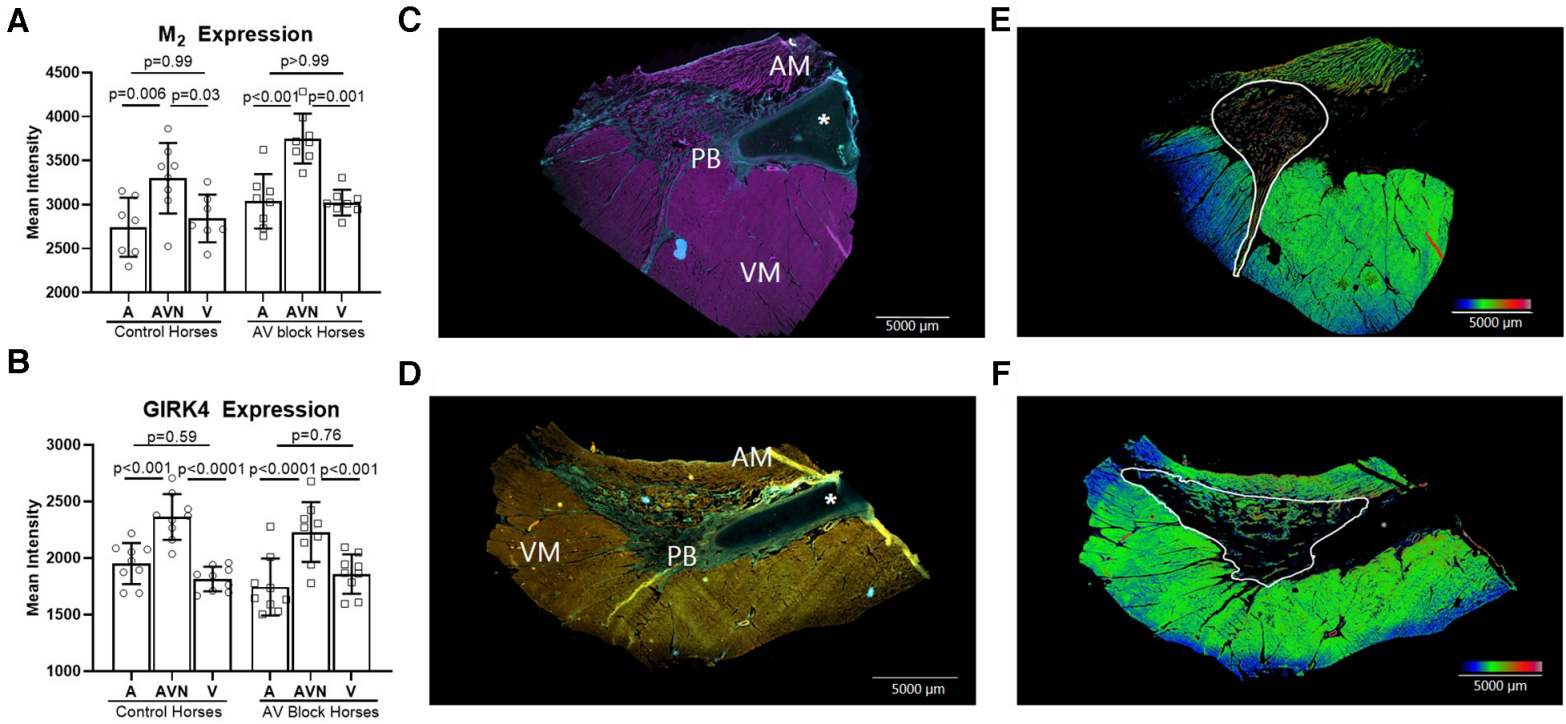
M_2_ receptor and GIRK4 expression in the atrium, AV node and the ventricle. The expression of M_2_ (*n* = 8 + 8)* (**A**) and GIRK4 (*n* = 9 + 9) (**B**) was significantly higher in the AV node (CN and PB together) compared to the atrium and ventricle in both groups, whereas no difference in the expression was found between the atria and the ventricles. (**C**) M_2_ positive immunohistochemical labeling of the atrium, the PB and the ventricle, where pink represents M_2_ positive areas and blue represents collagen. (**D**) GIRK4 positive immunohistochemical labeling of atrium, PB and ventricle, where yellow is GIRK4 positive areas, green is collagen. All data is presented as mean ± SD. (**E,F**) Heat maps demonstrating the different expression levels of M_2_ and GIRK4 respectively. The heat maps were created in ImageJ applying the “rainbow heat map” function, on segmented images where the background and collagen had been removed. The increase in the red spectrum in the AV nodal area signifies higher expression levels than in the surrounding atrium and ventricle. AM, atrial myocardium; AV, Atrioventricular; PB, Penetrating bundle; VM, Ventricular myocardium. An asterisk marks the aortic valve base of the non-coronary leaflet, which is cartilaginous in some horses. * from one horse, only the AV node was present on the slides, therfore atrial and ventricular analysis were missing from this horse.

The expression level of the M_2_ receptor was significantly higher in the AV block group in the PB (AV block group: *n* = 8, control group: *n* = 6) and in the total AV nodal expression (all sites averaged; *n* = 8 + 8) compared to the control group (*p* = 0.03 and *p* = 0.02, respectively; Figure 4A). No difference in the expression level of M_2_ in the CN was found between groups (AV block group: *n* = 6, Control: *n* = 6, *p* = 0.54). The expression of GIRK4 did not differ between groups (*n* = 9 + 9; Figure 4B). The expression level of both the M_2_ receptor and GIRK4 was significantly higher in the AV node (CN and PB combined) in both groups compared to the atria and ventricles (Figures 5A,B). In the ventricles the expression of GIRK4 was lower in areas remote to the AV node compared to areas close to the AV node (*p* = 0.01 and *p* = 0.005 for control horses and AV block horses, respectively). The difference in protein expression of M_2_ and GIRK4 in the different regions are illustrated in heat maps in Figures 5C,F.

A slight, but insignificant, correlation was found for M_2_ and the burden of second-degree AV block (*R*^2 ^= 0.19, *p* = 0.09) whereas GIRK4 and the number of second-degree AV block did not correlate at all (*R*^2 ^= 0.003, *P* = 0.83). The training status of the horses is highlighted in the plots and did not appear to correlate with the expression level either (Supplementary Figure S1).

## Discussion

### The m_2_R-I_k,ACh_ pathway and second-degree AV block in horses

Here, we demonstrate the involvement of the m_2_R-*I*_K,ACh_ pathway in second-degree AV blocks in horses. Furthermore, we identified an increased expression of the M_2_ receptors within the AV node in horses with a high burden of second-degree AV block episodes, perhaps constituting the underlying explanation for why some horses experience second-degree AV block in the setting of physiological (basal) ACh concentrations.

In all horses, the PR interval shortened and second-degree AV blocks were completely abolished under the influence of atropine, strongly indicating that m_2_R-*I*_K,ACh_ plays a substantial role in the AV nodal conduction in otherwise healthy horses, which is in agreement with previous studies (15, 18). The presence of frequent second-degree AV block episodes was not related to an increased expression of GIRK4 channels, which was equally expressed in the AV node of all horses. Instead, an increased expression of the M_2_ receptor was discovered, where the difference was more pronounced in the lower nodal tract in horses that presented with a high burden of second-degree AV block. This suggest that a different local expression of the M_2_ receptor exist, and may be a reasonable explanation for the very different burden of second-degree AV block episodes observed in horses. This is emphasised by the fact that atropine abolishes AV block in horses regardless of the AV block burden. Although, an increase in the expression level of M_2_ receptors was detected, it is unknown whether this directly translates into an increase in the activity of the receptor. At this point we are unable to study the functional role of the receptors in the equine AV node and further studies are required to clarify their activity. Unfortunately, a few samples were missing in the M_2_ analysis, resulting in the lack of data from two horses (one from each group) which might have influenced the observed difference in M_2_ expression. Additionally, the challenge of identifying antibodies specific to equine receptors represents a notable limitation in this study. However, we addressed this concern by conducting slice processing and imaging without primary antibodies as negative controls. As a result, we have confidence in the specificity of the antibodies utilized. Recent studies have detected the presence of other muscarinic subtypes, such as M_1_ and M_3_, in the heart of various mammals, including humans (19, 29–31). The impact of these muscarinic receptors on cardiac conduction has not been thoroughly investigated. However, it is plausible that these receptors may also exist in the equine heart and AV node, potentially exerting significant effects on electrical conduction. More studies in this area are needed.

In human patients with second-degree AV block, an increased cardiac fibrotic infiltration in the AV node region or bundle branches is often identified and proves to be the pathologic explanation for the arrhythmia (32). In our study, we also looked into the level of fibrotic content within the AV nodal area based on the histology staining (data not shown). We did not detect any differences in horses with AV block compared to the horses without, and we do not believe that increased fibrosis facilitates the higher number of second-degree AV block in healthy horses. In addition, this study did not include His bundle recordings. Thus we are unable to exclude the presence of infra-nodal AV block which in humans often signifies disease-related conduction disturbances (33). Among the horses with second-degree AV blocks included in this study, most exhibited PR interval prolongation preceding the AV block at all times, suggestive of Mobitz type I AV block. However, the definitive confirmation of this would necessitate His signal recordings. It is noteworthy that we previously conducted His signal recordings in a horse with PR prolongation and second-degree AV block, revealing that the AV conduction delay was situated in the His bundle (5). Indeed, further research is required to comprehensively understand the precise location of the AV conduction delay in horses.

Atrioventricular conduction is complex and the interplay between other ion channels and membrane transporters may also be influenced by parasympathetic activity and could be different among horses. Furthermore, despite not detecting a higher expression of GIRK4 in the AV node of the horses presenting with numerous second-degree AV block episodes, the functional role of the I_KACh_ channels may be different in these animals. In fact, it has previously been shown that in human patients suffering from atrial fibrillation, the expression of the Kir1.3 and Kir3.4, which constitute the I_KACh_ channel, were down-regulated, whilst the open probability was upregulated in atrial cardiomyocytes from humans undergoing cardiac surgery (34). Based on that data and the behavour of these cells they appeared to be agonist independent constitutively active which implies that the closing of the channels may be irresponsive to the blocking effect of the M_2_ receptor with atropine (34). While the activity of the channels in theory could be upregulated in animals with high degree of AV blocks, the effect of atropine in our study suggests that this may be less likely, as the AV block disappeared under the influence of atropine.

Yamaya et al. discovered that the PR interval was prolonged in horses with AV block compared to horses without, after blockage of the ANS during atrial pacing. This suggests that other factors not regulated by parasympathetic activity may also explain the difference in the AV block burden in horses (18). This lack of insight stresses that a more comprehensive analysis involving a larger panel of ion channels and membrane transporters should be studied in order to understand AV block origin in horses. We did not perform pacing-based electrophysiological studies on our horses during ANS block. The PR interval was similar in our horses after ANS block, but we do not know whether this parameter would have changed, had we used pacing protocols, as a possible rate-dependent effect on PR interval exist. The average HR between the two groups at the time of PR measurement was comparable. Therefore, it is unlikely that any potential rate-dependent effect on PR interval, if this exists, would have masked a difference.

We have previously shown that training induces slowing of the AV conduction in Standardbred racehorses (5, 15) and furthermore, that this was related to an intrinsic downregulation of ion channels facilitating the depolarisation of AV nodal cells, not due to changes in vagal activity (15). As both trained and untrained horses were included in the current study, we investigated if training could explain the increase in AV block and the expression of M_2_ receptor. Based on linear regression it appears that the burden of second-degree AV block has a slight correlation with the expression level of M_2_ regardless of training status. This might be accounted for by local differences in the parasympathetic pathway that could exist between horses, regardless of training status.

### The expression of the GIRK4 in the ventricles

In all horses, the expression of GIRK4 was significantly higher in the AV node compared to both the atrium and the ventricle. There was no difference in the atrial and the near nodal ventricular expression of GIRK4, which was surprising, as the I_KACh_ channel has been shown to be much more abundant in the atria compared to ventricles in mouse, rat, ferret and guinea pigs (35, 36). In horses, the gene expression of KCNJ5 encoding Kir3.4/GIRK4 has been shown to be under the detection level in ventricular tissue but highly abundant in atrial tissue (13). In a recent study, investigation of the mRNA level of KCNJ5 in human hearts revealed an equal expression of this gene in the left ventricle and the atria, suggesting that I_KACh_ may not be as atria specific as initially considered (37). The finding of GIRK4 expression in the ventricles of horses is in line with the functional data obtained by Fenner et al. who reported a transient QTc prolongation in horses when blocking the I_KACh_ channel (14). However, as we analysed the expression level of the GIRK4 in a more remote region in the ventricular septum from the AV node, a significantly lower expression of GIRK4 was detected. This implies that the expression of the I_KACh_ channel in the ventricles may be region specific, which would also explain why most studies hardly identify the expression of the GIRK4 channels in ventricular samples (13, 35, 36). It is unlikely, that the previously investigated biopsies in rat, ferrets, guinea pigs and horses (13, 36), were obtained in proximity to the atrioventricular junction, which may therefore explain the discrepancies between studies.

## Conclusion

In this study we showed that the m_2_R-*I*_K,ACh_ pathway is important in the genesis of second-degree AV block in Standardbred racehorses. In horses with a high burden of second-degree AV block an increased expression of the M_2_ receptor was identified in the AV node. No difference in the expression of GIRK4 was found in horses with second-degree AV block compared to horses without. A ventricular gradient in the expression of the GIRK4 was found where GIRK4 was equally expressed in the atria and in the near nodal region of the ventricles. However, this expression was significantly lower in ventricular regions more remote to the AV node. Furthermore, the findings in our study may have clinical relevance as we showed that a high burden of second-degree AV block is driven by parasympathetic activity and is not associated with an increased level of fibrosis. However, this study did not differentiate between AV nodal and infra-nodal conduction blocks, and further research is needed in order to fully understand the mechanisms underlying second-degree AV blocks in horses.

## Data Availability

The original contributions presented in the study are included in the article/**Supplementary Material**, further inquiries can be directed to the corresponding author.
