# Supplementary figures and images for: Muscarinic acetylcholine receptors M2 are upregulated in the atrioventricular nodal tract in horses with a high burden of second-degree atrioventricular block
Source: Front Cardiovasc Med. 2023 Nov 16;10:1102164. doi: 10.3389/fcvm.2023.1102164 (PMC10687567; doi:10.3389/fcvm.2023.1102164)

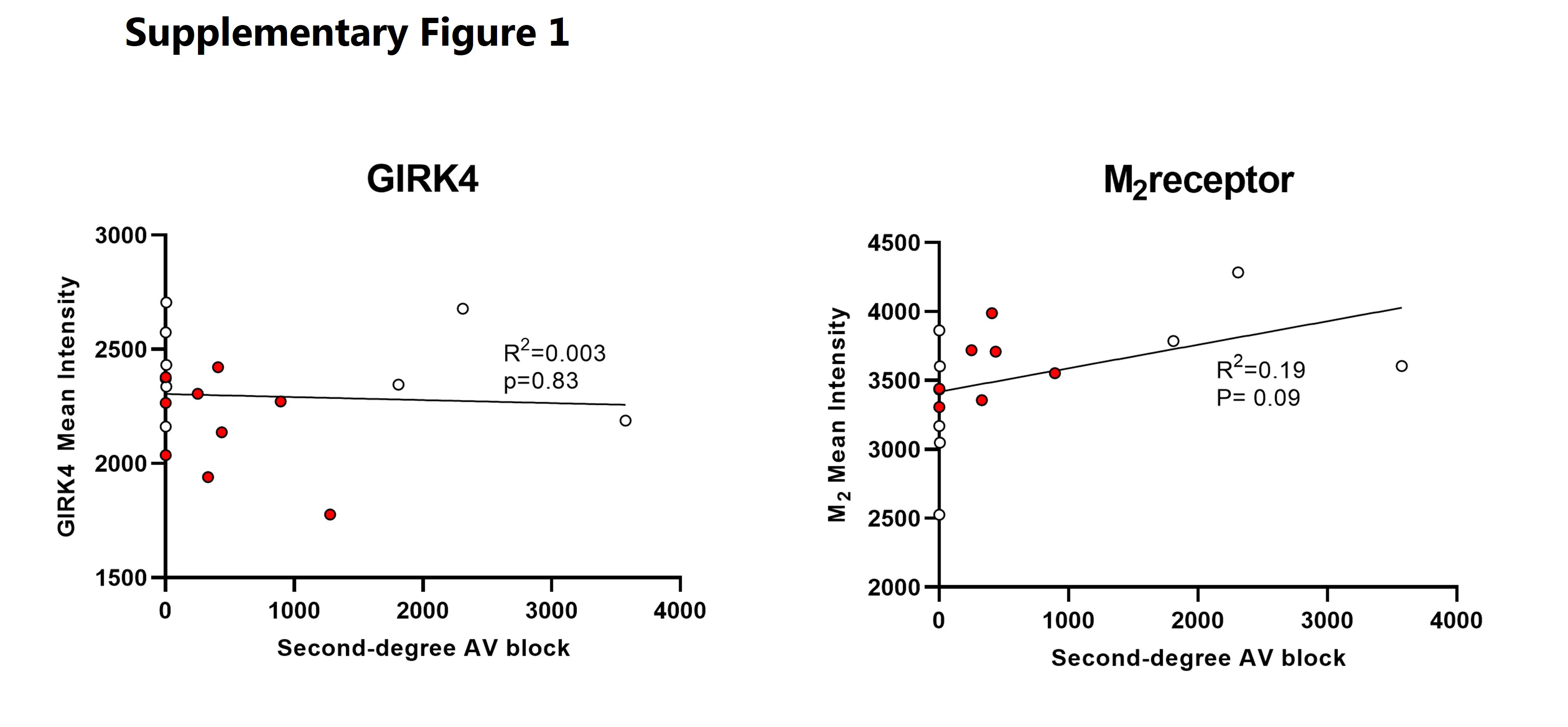

Supplement: Supplementary file 1 [file Image1.jpg]
