# Supplementary material for: Muscarinic acetylcholine receptors M2 are upregulated in the atrioventricular nodal tract in horses with a high burden of second-degree atrioventricular block
Source: Front Cardiovasc Med. 2023 Nov 16;10:1102164. doi: 10.3389/fcvm.2023.1102164 (PMC10687567; doi:10.3389/fcvm.2023.1102164)
Supplement: Supplementary file 2 [file Presentation1.pdf]

Supplemental material

Complete outline of the inclusion process and previous publications from the horses.

The 35 horses included in the current study were bought and dedicated to research with obtained owner consent and appropriate animal licenses.

The horses were housed at the Large Animal Teaching Hospital at the University of Copenhagen. The horses were housed in individual stalls at nighttime and grouped at paddocks at daytime. The horses were attended by a veterinarian each day during the study period.

The selection process for the current study was as following:

Baseline ECGs were collected from 35 horses that were purchased for research purposes. The ECGs obtained from these horses have been used in another publication looking into the training effect of exercise on ECG parameters. This was combined with ECGs from more than 200 client-owned horses and has been published [1].

Of these 35 horses, blocking of the autonomic nervous system had been performed in 30 horses. The tissue sampling and analysis were performed in a subset of these 35 horses. The horses were selected based on whether they had an extremely high number of second-degree AV block or none or very few second-degree AV block. This was done blinded to any other information on the horses, which led to the selection of 18 horses. These 18 horses included the tissue from six horses that had been included in a previous study looking into the effect of exercise on ion channel expression in the AV node [2].

Unfortunately, the already used samples led to the lack of samples from the entire AV node from two horses (one from each group) for the M_2 analysis and furthermore, we missed three samples from the compact AV node and two samples from the penetrating bundle for M_2 analysis.

Wenckebach measurements were successfully performed in 16 horses (attempted in 22). The measurements were performed in relation to another study focusing on epicardial mapping of the atria [3]. The procedure included hours of open chest procedure in horses under general anesthesia. The mapping was time-consuming and for some horses, the Wenckebach measurements were not performed. The Wenckebach measurements are not published elsewhere.

References

1. Nissen, S.D., et al., *Electrocardiographic characteristics of trained and untrained standardbred racehorses.* J Vet Intern Med, 2022. **36**(3): p. 1119-1130.
2. Mesirca, P., et al., *Intrinsic Electrical Remodeling Underlies Atrioventricular Block in Athletes.* Circ Res, 2021. **129**(1): p. e1-e20.
3. Carstensen, H., et al., *Long-Term Training Increases Atrial Fibrillation Sustainability in Standardbred Racehorses.* J Cardiovasc Transl Res, 2023.
